# Long-term effects of COVID-19 infection on bone mineral density

**DOI:** 10.7189/jogh.14.05029

**Published:** 2024-10-18

**Authors:** Zhan Wang, Zilong Li, Yechao Shen, Shengjun Qian, Mengling Tang, Jiaming He, Haoda Lu, Ning Zhang

**Affiliations:** 1 Department of Orthopaedic Surgery, The Second Affiliated Hospital, Zhejiang University School of Medicine, Zhejiang, China; 2 Orthopaedics Research Institute of Zhejiang University, Zhejiang, China; 3 Key Laboratory of Motor System Disease Research and Precision Therapy of Zhejiang Province, Zhejiang, China; 4 Zhejiang Provincial Clinical Medical Research Centre for Motor System Diseases, Zhejiang, China; 5 International Chinese Musculoskeletal Research Society, Zhejiang, China; 6 Department of Epidemiology and Biostatistics, Zhejiang University School of Public Health, Zhejiang, China; 7 First Affiliated Hospital of Xian Jiaotong University, Shaanxi, China; 8 Bioinformatics Institute, A*STAR, Singapore

## Abstract

**Background:**

In this study, we aimed to identify bone mineral density (BMD) trajectories of hospitalised patients with coronavirus disease 2019 (COVID-19) and to determine the prognostic role of the trajectory groups.

**Methods:**

This is a retrospective study of hospitalised patients with COVID-19 treated in our hospital from November 2022 to February 2023. BMD was manually measured from the thoracic 12 (T12) and lumbar one (L1) vertebra using chest computed tomography images. We constructed group trajectory models using group-based trajectory modelling. We performed the logistic regression analysis to associate the BMD trajectory pattern with clinical outcomes.

**Results:**

This study included 1767 patients. The mean follow-up time after discharge was 181.5 days (standard deviation (SD) = 9.7). There were 1137 (64.3%) male patients, and more than 80% of patients were aged >60 years. We successfully identified three latent BMD trajectories to reveal the dynamic effects of COVID-19 infection on bone health in patients, namely, the early low-normal decline group, the average, and the early high-rapid decline group. All groups demonstrated consistent overall declining trends. A significant association was observed between BMD trajectory pattern (T12 or L1) and baseline characteristics of sex, age, and penetrating keratoplasty (*P* < 0.05). Our study showed that the BMD trajectories were significantly associated with mortality. Furthermore, we found that these trajectories were also associated with the length of hospital stay.

**Conclusions:**

This study provided evidence for the COVID-19 process to bone health, as well as evidence on strengthening bone health management before and after COVID-19 infection. BMD trajectories may help manage bone health and guide treatment in patients with COVID-19.

Coronavirus disease 2019 (COVID-19) is caused by severe acute respiratory syndrome coronavirus two infection (SARS-CoV-2), which is rapidly becoming a global epidemic and poses a serious threat to human life and health [1]. While numerous studies have described the effects of COVID-19 on the nerves, lungs, kidneys, blood vessels, and heart, the musculoskeletal effects of COVID-19 have only recently attracted attention, particularly on bone metabolism, such as bone loss [2]. Recent studies have suggested that COVID-19 illness and treatment significantly negatively affect bone health and decrease hospitalised patients’ bone mineral density (BMD) [3–5]. Moreover, COVID-19 illness, treatment, and the patient’s factors are related to osteoporosis [6]. Meanwhile, BMD is closely related to the clinical course and prognosis of COVID-19 patients [7,8]. However, rare studies have been reported on the dynamic changes of BMD and its prognostic value in patients with COVID-19.

An international cohort study showed that about 24% of respondents with long-term COVID-19 reported bone ache or burning, with symptoms lasting up to seven months after the onset of COVID-19 [9]. Since the skeletal system expresses angiotensin-converting enzyme two receptors, which are used by SARS-CoV-2 to enter human cells [2], it is reasonable to believe that SARS-CoV-2 may impact bone metabolism. One recent study performed with hamsters showed that SARS-CoV-2 causes significant loss of bone trabeculae as early as four days post-infection. Furthermore, by day 60, the hamsters had recovered from the SARS-CoV-2 infection, but their bone density did not recover and even gradually decreased [10]. This suggested that bone loss extends from SARS-CoV-2 infection to recovery. Pathological bone loss due to COVID-19 infection may be an important but overlooked complication. However, there is a lack of studies from patients on the impact of the COVID-19 pandemic on long-term bone health.

Given the possibility that COVID-19 can cause long-term health complications, commonly known as long COVID [11], an immediate research priority is to investigate the BMD in these patients and to understand how it evolves. To provide new ideas and guidance for the treatment of osteoporosis and strengthening bone health management during the COVID-19 pandemic and future outbreaks, we reported the dynamic changes of BMD in patients with COVID-19. Furthermore, we developed and applied a group-based trajectory model to distinguish hospitalised individuals with COVID-19 by changes in BMD over time. We hypothesised that patients diagnosed with COVID-19 will exhibit distinct longitudinal trajectories of bone mineral density changes following infection, and these trajectories will be associated with varying clinical outcomes, such as mortality rates, length of hospital stay, and intensive care unit (ICU) admissions.

## METHODS

### Study population

**Figure 1:**
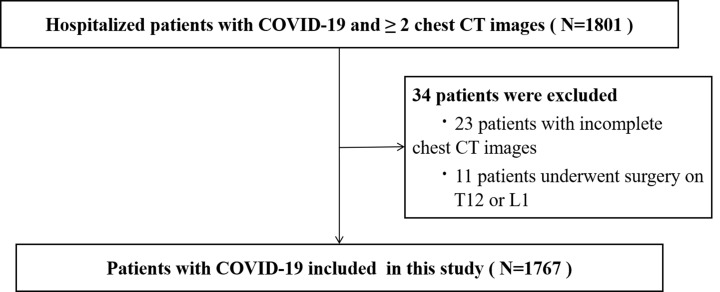
The flowchart for the selection of the study population. COVID-19 – coronavirus disease 2019. CT – computed tomography.

We retrospectively analysed the clinical information and chest high-resolution computed tomography (CT) data of hospitalised patients with COVID-19 treated in our hospital from November 2022 to February 2023. All patients had at least two chest CT scans. Real-time reverse-transcriptase polymerase chain reaction analysis was performed on nasal and/or throat swab specimens to diagnose COVID-19 infection. The diagnosis of COVID-19 infection was further supported by clinical manifestations, including pertinent signs and symptoms and radiographic findings suggestive of COVID-19 pneumonia [7]. Inclusion and exclusion criteria are shown in **Figure 1**. This study was reviewed and approved by the ethics committee from the local hospital (approval number 2023-0102).

### Data collection

We collected data using standardised case report forms or directly through patient interviews. The data manager and clinicians verified accuracy. Patients’ basic information included sex, age, admission to the ICU during hospitalisation, need for mechanical ventilation (MV), and percutaneous kyphoplasty (PKP) history. Clinical outcomes included mortality, length of ICU stay, and length of hospital stay.

### BMD measurement

Dual-energy x-ray absorptiometry (DXA) is the current standard for assessing BMD. As our study is retrospective, quantitative CT and DXA were not performed. Many studies have shown that the CT-based Hounsfield unit (HU) values of the bone trabecula can be used to evaluate osteoporosis [12], including the lumbar spine [13–15], cervical spine [16,17], thoracic spine [18], femur [19], pelvis [20], and ulna [21]. HU values of the spine measured by chest CT can be used to predict osteoporosis without additional cost and radiation exposure. Thus, chest CT HU can be an alternative for DXA [12,22]. Recently, one study supported the significance of HU values from chest CT for BMD assessment [23].

We made full use of chest CT to measure the HU values of thoracic 12 (T12) and lumbar one (L1) vertebra (Figure S1 in the **Online Supplementary Document**). The vertebral BMD was measured in a bone window (window position: 300, window width: 1600 HUs) on a non-contrast CT. All measurements were made in axial sections. An incomplete vertebral scan or a fractured vertebra was ignored. Two experienced orthopaedists measured vertebral BMD. The changes in vertebral HU values were dynamically monitored to reflect the dynamic changes of BMD.

The time of the patient’s first CT examination was recorded as day zero. To facilitate statistical analysis, data were processed according to the following rules: days 0–1 were recorded as day zero; days 2–4 as day three; days 5–7 as day six; days 8–10 as day nine; days 11–13 as day 12, and days 14–16 as day 15. Then, 30 days were used as a cycle. Days 17–45 were recorded as day 30, days 46–75 as day 60, and so on. The final statistical time from day 206 to day 225 was recorded as day 210. Each patient has performed at least two CT scans. The number of patients who underwent CT scans at different time points is shown in Table S1 in the **Online Supplementary Document**.

### Group-based trajectory modelling

Group-based trajectory modelling was achieved by using the Statistical Analysis System Trajectory Procedure process step-based approach. Group trajectory models were constructed using model fit effect metrics combined with professional interpretability. The model fit effectiveness metrics include average posterior probability, proportions per class, Bayesian information criterion, Bayesian information criterion difference between the two models and relative entropy. First, we fitted linear, square and cubic trajectory models for 2–6 groups. By comparing the fitting metrics of different models and the professional interpretability of the model trajectory patterns, we chose three cubic groups of trajectories. If not specified, the test level α is set to 0.05.

### Statistical analysis

We used SAS, version 9.4 for Windows (SAS Institute, Cary, North Carolina, USA) for data management and statistical analysis. We described qualitative information using n (%), and comparisons between groups were made using the χ^2^ test and Fisher exact probability method.

We used univariate logistic regression analysis to compare the risk of different clinical outcomes across trajectory groups, and we reported results as odds ratios (ORs) and 95% confidence intervals (CIs). We included patients’ basic information (sex, age, and comorbidity) and BMD trajectory patterns for multivariate logistic regression analysis. A *P*-value <0.05 was considered statistically significant.

## RESULTS

### Baseline characteristics between trajectory groups

A total of 1767 hospitalised patients diagnosed with COVID-19 were finally included in the study. Of all patients, 64.3% (n = 1137) were male, and the mean age was 73.2 years (standard deviation (SD) = 13.9). More than 80% of patients were aged >60 years. The average hospital stay for all patients was 12.7 days. 315 patients were admitted to ICU, and 325 patients received MV treatment. There were 42 (2.4%) patients who had undergone PKP surgery before COVID-19 infection. The average follow-up time after discharge was 181.5 days (SD = 9.7) (**Table 1**, **Table 2**).

**Table 1:** Baseline characteristics of patients in different trajectory groups based on T12 BMD*

		BMD trajectory pattern (T12)			
**Characteristics**	**All (n = 1767)**	**Early low-normal decline (n = 634)**	**Average (n = 844)**	**Early high-rapid decline (n = 289)**	***P*-value**
Male	1137 (64.3)	339 (53.5)	584 (69.2)	214 (74.1)	<0.001†
Age in years, x̄ (SD)					
*≤40*	48	1 (0.2)	9 (1.1)	38 (13.2)	<0.001†
*40–60*	264	24 (3.8)	131 (15.5)	109 (37.7)	
*>60*	1455	609 (96.1)	704 (83.4)	142 (49.1)	
ICU	315 (17.8)	115 (18.1)	154 (18.3)	46 (15.9)	0.649
MV	325 (18.4)	123 (19.4)	155 (18.4)	47 (16.3)	0.521
PKP	42 (2.4)	17 (2.7)	31 (2.8)	1 (0.4)	0.045†
Comorbidity	599 (33.9)	221 (34.9)	294 (34.8)	84 (29.1)	0.169
Anti-osteoporosis treatment	131 (7.4)	50 (7.9)	56 (6.6)	25 (8.7)	0.447

BMD – bone mineral density, ICU – intensive care unit, MV – mechanical ventilation, PKP – percutaneous kyphoplasty, SD – standard deviation, T12 – thoracic 12, x̄ – mean

*Presented as n (%) unless specified otherwise.

†Statistically significant.

**Table 2:** Baseline characteristics of patients in different trajectory groups based on L1 BMD*

		BMD trajectory pattern (L1)			
**Characteristics**	**All (n = 1767)**	**Early low-normal decline (n = 589)**	**Average (n = 965)**	**Early high-rapid decline (n = 213)**	***P*-value**
Male	1137 (64.3)	315 (53.5)	660 (68.4)	162 (76.1)	<0.001†
Age in years, x̄ (SD)					
*≤40*	48	0 (0.0)	19 (2.0)	29 (13.6)	<0.001†
*40–60*	264	21 (3.6)	162 (16.8)	81 (38.0)	
*>60*	1455	568 (96.4)	784 (81.2)	103 (48.4)	
ICU	315 (17.8)	103 (17.5)	177 (18.3)	35 (16.4)	0.777
MV, n (%)	325 (18.4)	110 (18.7)	180 (18.7)	35 (16.4)	0.733
PKP	42 (2.4)	11 (1.9)	31 (3.2)	0 (0.0)	0.013†
Comorbidity	599 (33.9)	214 (36.3)	316 (32.7)	69 (32.4)	0.310
Anti-osteoporosis treatment	131 (7.4)	46 (7.8)	67 (6.9)	18 (8.5)	0.684

BMD – bone mineral density, ICU – intensive care unit, L1 – lumbar one, MV – mechanical ventilation, PKP – percutaneous kyphoplasty, SD – standard deviation, x̄ – mean

*Presented as n (%) unless specified otherwise.

†Statistically significant.

**Figure 2:**
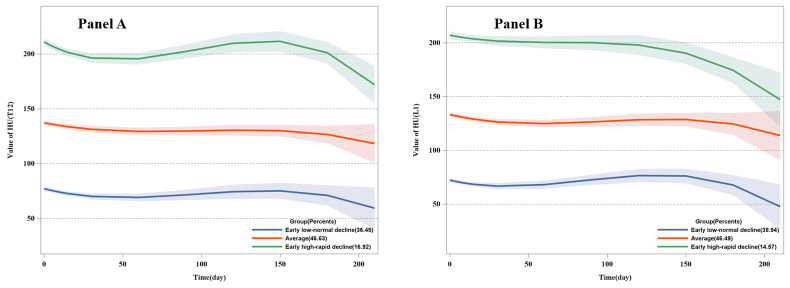
Three trajectory patterns were determined. **Panel A.** By T12 BMD. **Panel B.** By L1 BMD. HU – Hounsfield units, L1 – lumbar one, T12 – thoracic 12.

**Figure 3:**
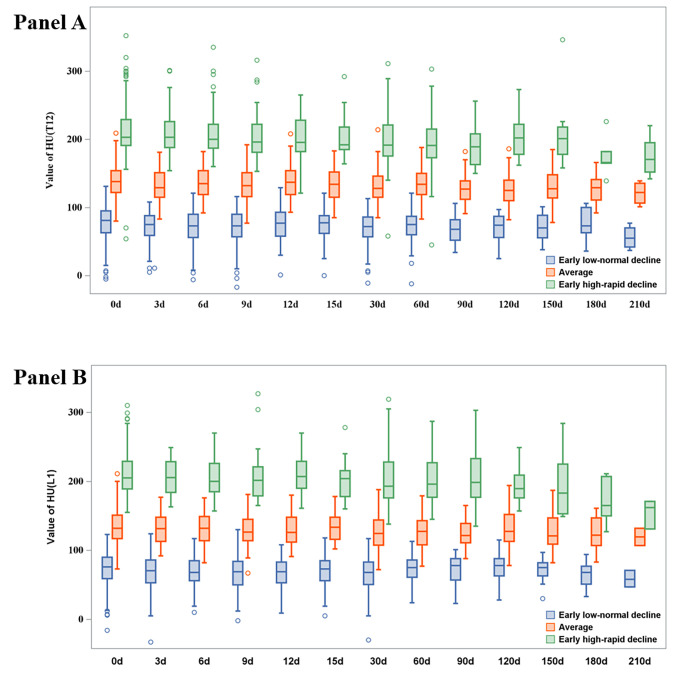
Cumulative BMD in the three trajectory groups. There were statistical differences in BMD among the three groups at each time point. **Panel A.** By T12. **Panel B.** By L1. HU – Hounsfield units, L1 – lumbar one, T12 – thoracic 12.

We successfully identified three latent BMD trajectories to reveal the dynamic effects of COVID-19 infection on bone health in patients (**Figure 2**). Significant differences in BMD among the three trajectory groups at each time point were observed (**Figure 3**). BMD trajectory one, described as an early low-normal decline group, showed subjects with low HU values on the first day, followed by a gradual decline, with the final result remaining lower than the initial HU values. BMD trajectory two, described as the average group, showed that subjects initially maintained a moderate BMD, which decreased over time but not significantly. BMD trajectory three, described as the early high-rapid decline group, presents subjects starting with high HU values, which slowly declined over time and then dropped significantly below the initial HU values. One typical case of the early high-rapid decline group is shown in Figure S2 in the **Online Supplementary Document**. Ultimately, all groups demonstrated consistent overall declining trends when juxtaposed with their initial HU values. Additionally, BMD changes in the entire population and in three BMD trajectories are presented (Figures S3–4 in the **Online Supplementary Document**).

A significant association was observed between BMD trajectory pattern (T12 or L1) and baseline characteristics of sex, age, and PKP (*P* < 0.05). The proportion of males in the early high-rapid decline group was higher than in the early low-normal decline group or average group. The proportion of age >60 years in the early low-normal decline group was significantly higher than that in an average group or early high-rapid decline group. The proportion of PKP in the early high-rapid decline group was significantly lower than in an average or low-normal decline group. ICU, MV, comorbidity, and anti-osteoporosis treatment were not associated with BMD trajectory patterns (T12 or L1) (**Table 1**, **Table 2**).

### Clinical outcomes

There were 213 (12.1%) patients who died during the follow-up. 889 (50.3%) were hospitalised for more than 10 days, and 132 (7.5%) were admitted to the ICU for more than 10 days. Additionally, eight of the 42 patients (19.0%) who had undergone PKP surgery died. A significant association was observed between BMD trajectory pattern (T12 or L1) and clinical outcomes of mortality and length of hospital stay (*P* < 0.05). Length of ICU stay was not associated with BMD trajectory pattern (T12 or L1) (**Table 3**, **Table 4**).

**Table 3:** Clinical outcomes of the patients with different BMD trajectory patterns (T12)*

		Bone density trajectory pattern (T12)			
**Characteristics**	**All (n = 1767)**	**Early low-normal decline (n = 634)**	**Average (n = 844)**	**Early high-rapid decline (n = 289)**	***P*-value**
Mortality	213 (12.1)	91 (14.4)	99 (11.7)	23 (8.0)	0.020†
Hospital stay in days					
*≤10*	878 (49.7)	284 (44.8)	429 (50.8)	165 (57.1)	0.002†
*>10*	889 (50.3)	350 (55.2)	415 (49.2)	124 (42.9)	
ICU stay in days					
*0*	1452 (82.2)	519 (81.9)	690 (81.7)	243 (84.1)	0.198
*1–10*	183 (10.3)	65 (10.2)	84 (10.0)	34 (11.8)	
*>10*	132 (7.5)	50 (7.9)	70 (8.3)	12 (4.1)	

BMD – bone mineral density, ICU – intensive care unit, T12 – thoracic 12

*Presented as n (%).

†Statistically significant.

**Table 4:** Clinical outcomes of the patients with different BMD trajectory patterns (L1)*

		Bone density trajectory pattern (L1)			
**Characteristics**	**All (n = 1767)**	**Early low-normal decline (n = 589)**	**Average (n = 965)**	**Early high-rapid decline (n = 213)**	***P*-value**
Mortality	213 (12.1)	85 (14.4)	111 (11.5)	17 (8.0)	0.034†
Hospital stay in days					
*≤10*	878 (49.7)	276 (46.9)	476 (49.3)	126 (59.2)	0.008†
*>10*	889 (50.3)	313 (53.1)	489 (50.7)	87 (40.8)	
ICU stay in days					
*0*	1452 (82.2)	486 (82.5)	788 (81.6)	178 (83.6)	0.638
*1–10*	183 (10.3)	56 (9.5)	103 (10.7)	24 (11.3)	
*>10*	132 (7.5)	47 (8.0)	74 (7.7)	11 (5.1)	

BMD – bone mineral density, ICU – intensive care unit, L1 – lumbar one

*Presented as n (%).

†Statistically significant.

Based on T12 BMD, the mortality rate was significantly higher in the early low-normal decline group compared to the early high-rapid decline (14.4% vs 8.0%). The proportion of hospitalisations longer than 10 days was significantly higher in the early low-normal decline group than in the early high-rapid decline group (55.2% vs 42.9%). Similar results were seen based on L1 BMD (**Table 4**).

### Association of the BMD trajectory pattern with clinical outcomes

The existing data indicate possible clinical associations between COVID-19 infection and BMD. However, this retrospective study cannot establish causality. Based on T12 BMD, compared with the early low-normal decline group, the early high-rapid decline group was significantly associated with decreased mortality (OR = 0.52; 95% CI = 0.32–0.83). Compared with the early low-normal decline group, the average group and the early high-rapid decline group were significantly associated with a decreased risk of length of hospital stay of more than 10 days (OR = 0.78, 95% CI = 0.64–0.97; OR = 0.61, 95% CI = 0.46–0.81). In terms of length of ICU stay, no significant correlation was observed between the early low-normal decline group and the early high-rapid decline group (**Table 5**). Similar results were seen based on L1 BMD (**Table 6**). Additionally, multivariate logistic regression showed that BMD trajectory pattern was an independent risk factor for mortality or length of hospital stay (>10 days) (Tables S2–5 in the **Online Supplementary Document**).

**Table 5:** The odds ratio for risks of clinical outcomes by the three subgroups of different BMD trajectory patterns (T12)

Clinical outcomes (T12)	Events, n (%)	OR (95% CI)	*P*-value
Primary (mortality)			
*Early low-normal decline*	91 (14.35)	ref.	
*Average*	99 (11.73)	0.79 (0.58–1.08)	0.136
*Early high-rapid decline*	23 (7.96)	0.52 (0.32–0.83)	0.007*
Secondary (length of hospital stay)†			
*Early low-normal decline*	350 (55.2)	ref.	
*Average*	415 (49.2)	0.78 (0.64–0.97)	0.022*
*Early high-rapid decline*	124 (42.9)	0.61 (0.46–0.81)	<0.001*
Secondary (length of ICU stay)‡			
*Early low-normal decline*	50 (7.89)	ref.	
*Average*	70 (8.29)	0.99 (0.76–1.29)	0.932
*Early high-rapid decline*	12 (4.15)	1.21 (0.83–1.76)	0.323

BMD – bone mineral density, CI – confidence interval, ICU – intensive care unit, OR – odds ratio, ref – reference, T12 – thoracic 12

*Statistically significant.

†Event: length of hospital stay >10 days.

‡Event: length of ICU stay >10 days.

**Table 6:** The odds ratio for risks of clinical outcomes by the 3 subgroups of different BMD trajectory patterns (L1)

Clinical outcomes (L1)	Events, n (%)	OR (95% CI)	*P*-value
Primary (mortality)			
*Early low-normal decline*	85 (14.43)	ref.	
*Average*	111 (11.50)	0.77 (0.57–1.04)	0.092
*Early high-rapid decline*	17 (7.98)	0.51 (0.30–0.89)	0.017*
Secondary (length of hospital stay)†			
*Early low-normal decline*	313 (53.1)	ref.	
*Average*	489 (50.7)	0.91 (0.74–1.11)	0.345
*Early high-rapid decline*	87 (40.9)	0.61 (0.44–0.84)	0.002*
Secondary (length of ICU stay)‡			
*Early low-normal decline*	47 (7.98)	ref.	
*Average*	74 (7.67)	0.95 (0.73–1.24)	0.716
*Early high-rapid decline*	11 (5.16)	1.11 (0.73–1.69)	0.633

BMD – bone mineral density, CI – confidence interval, ICU – intensive care unit, L1 – lumbar one, OR – odds ratio, ref – reference

*Statistically significant.

†Event: length of hospital stay >10 days.

‡Event: length of ICU stay >10 days.

## DISCUSSION

Recently, extrapulmonary manifestations caused by long COVID-19 have gradually attracted attention. However, less is known about the skeletal consequences of COVID-19. We identified three latent BMD trajectories to reveal the dynamic effects of COVID-19 infection on bone health in patients, namely, the early low-normal decline group, the average group, and the early high-rapid decline group. Our study showed that the BMD trajectory pattern (T12 or L1) was significantly correlated with baseline characteristics of sex, age, and PKP (*P* < 0.05). Moreover, these trajectories were significantly associated with mortality and length of hospital stay. Our study provided preliminary evidence of the association between COVID-19 infection and BMD but cannot prove their causation.

In our study, the selection of a three-trajectory model of BMD was assessed based on several indicators. A good model fit is indicated by 1) average posterior probability >0.7 for each group, 2) proportions per class >5%, 3) Bayesian information criterion close to zero, 4) the large Bayesian information criterion difference between complex and simple models (the more complex models are accepted), and 5) relative entropy >0.8. According to the results, we chose the three-trajectory model (Tables S6–S7 in the **Online Supplementary Document**). Additionally, the fitting effect of each trajectory group in the three-trajectory model was also good (Tables S8–S9 in the **Online Supplementary Document**). A good model fit in the three-trajectory model was indicated as follows: 1) average posterior probability for each group >0.7, 2) the odds of correct classification for each group >5, and 3) a close correspondence between proportions per class and πj.

Our study showed the longitudinal effect of COVID-19 on BMD, which was measured by quantitative chest CT. The overall trends of BMD in those three latent trajectories were first decreased, then stable or gradually increased, and finally decreased again, showing an overall downward trend. There was one study that showed that BMD in 58 patients with COVID-19 decreased by 8.6% (SD = 10.5) on average 81 days (SD = 48) after discharge [6]. However, the number of patients in this study was very small, and follow-up was short. Therefore, one of the long-term effects of COVID-19 on bone health was BMD decrease.

A significant association was observed between BMD trajectory pattern and baseline characteristics of sex, age, and PKP. Our study showed that the prevalence of COVID-19 was associated with the male gender, which was similar to the results of previous studies [24]. The proportion of early low-normal decline group in female patients was significantly higher than that in male patients. This may be explained by the significantly higher incidence of bone loss or osteoporosis in women than men. The proportion of the early low-normal decline group in patients aged >60 years was higher than that in patients aged <60 years. This may be explained by the fact that BMD decreases with age. However, our analyses showed that ICU, MV, and anti-osteoporosis treatment were not associated with BMD trajectory patterns. This may be due to a lower incidence of positive events. As for comorbidity, we only counted whether patients had comorbidities and did not analyse the number and severity of comorbidities in depth. More research is needed to further clarify our findings.

BMD is a key prognostic factor for many benign and malignant diseases, such as coronary artery disease, chronic obstructive pulmonary disease, cerebrovascular disease, and breast cancer [25–27]. Recent studies have shown that vertebral fractures are highly prevalent during the COVID-19 pandemic, and lower BMD can predict a poorer prognosis for patients with COVID-19 [7,8]. However, the prognostic value of dynamic BMD among patients with COVID-19 has not been thoroughly studied. Our study showed that these latent BMD trajectories were associated with clinical outcomes. Compared with the early low-normal decline group, the early high-rapid decline group was associated with decreased mortality. Additionally, we found that the early high-rapid decline group was associated with a decreased risk of hospital stay of >10 days. Those results suggest that maintaining a high level of BMD after COVID-19 may be helpful to reduce mortality and hospital stay.

It is worth mentioning that the mortality rate of hospitalised patients with COVID-19 who had previously performed PKP surgery was higher than that of the overall hospitalised patients with COVID-19. As we all know, PKP surgeries have been widely used to treat osteoporotic vertebral compression fractures. Patients who undergo PKP often have osteoporosis and have poor bone density. Lower BMD was associated with higher mortality risk in patients with osteoporosis [28]. In the context of COVID-19, patients with PKP surgery might have very poor BMD status which may worsen their overall health outcomes. Active anti-osteoporosis treatment is crucial for osteoporosis patients during the COVID-19 pandemic. Despite the difficulties in managing osteoporosis during the COVID-19 pandemic, many scholars supported the continuation of anti-osteoporosis treatment [29,30]. Filippo et al. [31] reported that low vitamin D levels were correlated with long COVID-19. Furthermore, many studies showed that vitamin D has potential benefits for the COVID-19 pandemic and is being considered a possible prevention strategy for COVID-19 [32–34].

COVID-19 itself and its treatment, as well as patient factors, have been associated with bone loss. SARS-CoV2 infection can cause inflammatory activation via direct and indirect effects on osteoclasts, leading to severe bone loss during illness and recovery [3]. Glucocorticoids are used to treat severely ill patients with COVID-19, but they can cause bone loss [35]. Vitamin D is an immunomodulatory hormone with proven efficacy against various upper respiratory tract infections [36]. Patients with vitamin D deficiency may be at a higher risk of contracting COVID-19 and developing severe COVID-19 than patients without vitamin D deficiency, which may lead to a higher incidence of osteoporosis than patients with moderate COVID-19 [37]. A recent study showed that normal levels of endogenous vitamin D are associated with reduced severity and risk of unfavourable outcomes in COVID-19, possibly via attenuation of tissue-specific hyperinflammation [38]. Further, another study revealed that vitamin D regulated COVID-19-associated severity by suppressing the nucleotide oligomerisation domain, leucine-rich repeats, and pyrin domain-containing protein three inflammasome pathway [39]. Thus, vitamin D is expected to be one of the critical components for treating COVID-19 infection. Studies have shown that age and hypocalcaemia are also associated with bone loss in patients with COVID-19 [6,40]. Additionally, the BMD of patients with COVID-19 may decrease due to reduced activity or bed rest.

This study was the first to investigate the dynamic effect of BMD trajectories on clinical outcomes in patients with COVID-19. There are some limitations in the present study. First, due to the retrospective nature of this study, no causal relationship between BMD trajectories and clinical outcomes can be explained. Second, the time interval and frequency of chest CT re-examination in patients with COVID-19 are inconsistent, which may affect the accuracy and reliability of BMD measurements. Patients with few CT scans cannot accurately and dynamically reflect the BMD changes. In future studies, we suggest implementing a more standardised approach to CT scan timing, such as fixed intervals or specific time points for all patients. This would help reduce variability and potentially enhance the accuracy and reliability of the findings. Third, this study used an accessible chest CT to obtain BMD without using standard testing methods for BMD. Many previous studies reveal that lumbar CT HU can be an additional diagnostic tool for osteoporosis and an alternative for DXA [12,22]. Additionally, one recent study supports the significance of HU values from chest CT for BMD assessment [23]. In the future, we plan to conduct a prospective clinical trial and include DXA or quantitative CT as the methods to evaluate BMD. Fourth, this study lacked a control group, which may affect the validity of interpretation and inference of results. Additionally, the dynamic inflammatory status of patients was lacking, which may be relevant to the BMD trajectory pattern. Many studies showed that inflammation was negatively associated with the BMD [41–43]. The complex correlation between inflammation and BMD requires further evaluation in large prospective studies. Changes in inflammatory indicators in blood should be detected simultaneously with chest CT. Further, the trend of changes in inflammatory indicators (interleukin six, C-reactive protein, etc) combined with CT HU values should be analysed.

## CONCLUSIONS

In this study, we highlighted the contribution of the COVID-19 process to bone health and the need for better prevention and treatment of bone loss in patients before and after COVID-19 infection. BMD trajectories may help manage bone health and guide treatment in patients with COVID-19. However, we strongly believe that additional experimental and clinical data are needed to better understand the long-term effects of COVID-19 on BMD.

## Additional material


Online Supplementary Document

## References

[1] Li G Hilgenfeld R Whitley R De Clercq E Therapeutic strategies for COVID-19: progress and lessons learned. Nat Rev Drug Discov. 2023;22:449–75. 10.1038/s41573-023-00672-y 37076602 PMC10113999

[2] Disser NP De Micheli AJ Schonk MM Konnaris MA Piacentini AN Edon DL Musculoskeletal Consequences of COVID-19. J Bone Joint Surg Am. 2020;102:1197–204. 10.2106/JBJS.20.00847 32675661 PMC7508274

[3] Tsourdi E Hofbauer LC Rauner M The Impact of COVID-19 in Bone Metabolism: Basic and Clinical Aspects. Horm Metab Res. 2022;54:540–8. 10.1055/a-1825-9641 35419776

[4] Hu CL Zheng MJ He XX Liu DC Jin ZQ Xu WH COVID-19 and bone health. Eur Rev Med Pharmacol Sci. 2023;27:3191–200. 37070922 10.26355/eurrev_202304_31953

[5] Awosanya OD Dadwal UC Imel EA Yu Q Kacena MA The Impacts of COVID-19 on Musculoskeletal Health. Curr Osteoporos Rep. 2022;20:213–25. 10.1007/s11914-022-00734-x 35723777 PMC9207429

[6] Berktaş BM Gökçek A Hoca NT Koyuncu A COVID-19 illness and treatment decrease bone mineral density of surviving hospitalized patients. Eur Rev Med Pharmacol Sci. 2022;26:3046–56. 35503607 10.26355/eurrev_202204_28636

[7] di Filippo L Formenti AM Doga M Pedone E Rovere-Querini P Giustina A Radiological Thoracic Vertebral Fractures are Highly Prevalent in COVID-19 and Predict Disease Outcomes. J Clin Endocrinol Metab. 2021;106:e602–14. 10.1210/clinem/dgaa738 33159451 PMC7797741

[8] Tahtabasi M Kilicaslan N Akin Y Karaman E Gezer M Icen YK The Prognostic Value of Vertebral Bone Density on Chest CT in Hospitalized COVID-19 Patients. J Clin Densitom. 2021;24:506–15. 10.1016/j.jocd.2021.07.007 34353732 PMC8302819

[9] Davis HE Assaf GS McCorkell L Wei H Low RJ Re’em Y Characterizing long COVID in an international cohort: 7 months of symptoms and their impact. EClinicalMedicine. 2021;38:101019. 10.1016/j.eclinm.2021.101019 34308300 PMC8280690

[10] Qiao W Lau HE Xie H Poon VK Chan CC Chu H SARS-CoV-2 infection induces inflammatory bone loss in golden Syrian hamsters. Nat Commun. 2022;13:2539. 10.1038/s41467-022-30195-w 35534483 PMC9085785

[11] Davis HE McCorkell L Vogel JM Topol EJ Long COVID: major findings, mechanisms and recommendations. Nat Rev Microbiol. 2023;21:133–46. 10.1038/s41579-022-00846-2 36639608 PMC9839201

[12] Chen H Zhu X Zhou Q Pu X Wang B Lin H Utility of MRI-based vertebral bone quality scores and CT-based Hounsfield unit values in vertebral bone mineral density assessment for patients with diffuse idiopathic skeletal hyperostosis. Osteoporos Int. 2024;35:705–15. 10.1007/s00198-023-06999-x 38148381

[13] Pinto EM Neves JR Teixeira A Frada R Atilano P Oliveira F Efficacy of Hounsfield Units Measured by Lumbar Computer Tomography on Bone Density Assessment: A Systematic Review. Spine. 2022;47:702–10. 10.1097/BRS.0000000000004211 34468433

[14] Kim KH Kim TH Kim SW Kim JH Lee HS Chang IB Significance of Measuring Lumbar Spine 3-Dimensional Computed Tomography Hounsfield Units to Predict Screw Loosening. World Neurosurg. 2022;165:e555–62. 10.1016/j.wneu.2022.06.104 35772704

[15] Choi MK Kim SM Lim JK Diagnostic efficacy of Hounsfield units in spine CT for the assessment of real bone mineral density of degenerative spine: correlation study between T-scores determined by DEXA scan and Hounsfield units from CT. Acta Neurochir (Wien). 2016;158:1421–7. 10.1007/s00701-016-2821-5 27177734

[16] Han K You ST Lee HJ Kim IS Hong JT Sung JH Hounsfield unit measurement method and related factors that most appropriately reflect bone mineral density on cervical spine computed tomography. Skeletal Radiol. 2022;51:1987–93. 10.1007/s00256-022-04050-4 35419706

[17] Liang X Liu Q Xu J Ding W Wang H Hounsfield Unit for Assessing Bone Mineral Density Distribution Within Cervical Vertebrae and Its Correlation With the Intervertebral Disc Degeneration. Front Endocrinol (Lausanne). 2022;13:920167. 10.3389/fendo.2022.920167 35872993 PMC9304988

[18] Wang P She W Mao Z Zhou X Li Y Niu J Use of routine computed tomography scans for detecting osteoporosis in thoracolumbar vertebral bodies. Skeletal Radiol. 2021;50:371–9. 10.1007/s00256-020-03573-y 32767060

[19] Kılınc RM Açan AE Türk G Kılınç CY Yeniçeri İ Evaluation of femoral head bone quality by Hounsfield units: a comparison with dual-energy X-ray absorptiometry. Acta Radiol. 2022;63:933–41. 10.1177/02841851211021035 34078124

[20] Inagaki N Tanaka T Udaka J Akiyama S Matsuoka T Saito M Distribution of hounsfield unit values in the pelvic bones: a comparison between young men and women with traumatic fractures and older men and women with fragility fractures: a retrospective cohort study. BMC Musculoskelet Disord. 2022;23:305. 10.1186/s12891-022-05263-3 35351073 PMC8966165

[21] Wagner SC Dworak TC Grimm PD Balazs GC Tintle SM Measurement of Distal Ulnar Hounsfield Units Accurately Predicts Bone Mineral Density of the Forearm. J Bone Joint Surg Am. 2017;99:e38. 10.2106/JBJS.15.01244 28419040

[22] Courtois EC Davidson IU Ohnmeiss DD Guyer RD Evaluating alternatives to dual-energy x-ray absorptiometry for assessing bone quality in patients undergoing spine surgery. J Neurosurg Spine. 2023;40:84–91. 10.3171/2023.8.SPINE23450 37862719

[23] Xue C Sun G Wang N Liu X He G Wei Y Value of Hounsfield units measured by chest computed tomography for assessing bone density in the thoracolumbar segment of the thoracic spine. Asian Spine J. 2024;18:336–45. 10.31616/asj.2023.0438 38917853 PMC11222884

[24] Clement ND Ng N Simpson CJ Patton RFL Hall AJ Simpson A The prevalence, mortality, and associated risk factors for developing COVID-19 in hip fracture patients: a systematic review and meta-analysis. Bone Joint Res. 2020;9:873–83. 10.1302/2046-3758.912.BJR-2020-0473.R1 33350316 PMC9021904

[25] Hwang HJ Lee SM Seo JB Kim JE Choi HY Kim N Quantitative Vertebral Bone Density Seen on Chest CT in Chronic Obstructive Pulmonary Disease Patients: Association with Mortality in the Korean Obstructive Lung Disease Cohort. Korean J Radiol. 2020;21:880–90. 10.3348/kjr.2019.0551 32524788 PMC7289694

[26] Park SH Jeong YM Lee HY Kim EY Kim JH Park HK Opportunistic use of chest CT for screening osteoporosis and predicting the risk of incidental fracture in breast cancer patients: A retrospective longitudinal study. PLoS One. 2020;15:e0240084. 10.1371/journal.pone.0240084 33052943 PMC7556442

[27] Veronese N Stubbs B Crepaldi G Solmi M Cooper C Harvey NC Relationship Between Low Bone Mineral Density and Fractures With Incident Cardiovascular Disease: A Systematic Review and Meta-Analysis. J Bone Miner Res. 2017;32:1126–35. 10.1002/jbmr.3089 28138982 PMC5417361

[28] Hsu CS Chang ST Cheng YY Lee HT Chen CH Deng YL Low Bone Mineral Density and Calcium Levels as Risks for Mortality in Patients with Self-Discontinuation of Anti-Osteoporosis Medication. Int J Environ Res Public Health. 2021;19:197. 10.3390/ijerph19010197 35010457 PMC8750269

[29] Cromer SJ Yu EW Challenges and Opportunities for Osteoporosis Care During the COVID-19 Pandemic. J Clin Endocrinol Metab. 2021;106:e4795–e4808. 10.1210/clinem/dgab570 34343287 PMC8385842

[30] Yu EW Tsourdi E Clarke BL Bauer DC Drake MT Osteoporosis Management in the Era of COVID-19. J Bone Miner Res. 2020;35:1009–13. 10.1002/jbmr.4049 32406536 PMC7273005

[31] di Filippo L Frara S Nannipieri F Cotellessa A Locatelli M Rovere Querini P Low vitamin D levels are associated with Long COVID syndrome in COVID-19 survivors. J Clin Endocrinol Metab. 2023;108:e1106–16. 10.1210/clinem/dgad207 37051747 PMC10505553

[32] Charoenngam N Shirvani A Holick MF Vitamin D and Its Potential Benefit for the COVID-19 Pandemic. Endocr Pract. 2021;27:484–93. 10.1016/j.eprac.2021.03.006 33744444 PMC7965847

[33] Contreras-Bolívar V García-Fontana B García-Fontana C Muñoz-Torres M Vitamin D and COVID-19: where are we now? Postgrad Med. 2023;135:195–207. 10.1080/00325481.2021.2017647 34886758 PMC8787834

[34] Ebrahimzadeh A Mohseni S Narimani B Ebrahimzadeh A Kazemi S Keshavarz F Association between vitamin D status and risk of COVID-19 in-hospital mortality: A systematic review and meta-analysis of observational studies. Crit Rev Food Sci Nutr. 2023;63:5033–43. 10.1080/10408398.2021.2012419 34882024

[35] Chotiyarnwong P McCloskey EV Pathogenesis of glucocorticoid-induced osteoporosis and options for treatment. Nat Rev Endocrinol. 2020;16:437–47. 10.1038/s41574-020-0341-0 32286516

[36] Barrea L Verde L Grant WB Frias-Toral E Sarno G Vetrani C Vitamin D: A Role Also in Long COVID-19? Nutrients. 2022;14:1625. 10.3390/nu14081625 35458189 PMC9028162

[37] Sulli A Gotelli E Casabella A Paolino S Pizzorni C Alessandri E Vitamin D and Lung Outcomes in Elderly COVID-19 Patients. Nutrients. 2021;13:717. 10.3390/nu13030717 33668240 PMC7996150

[38] Renieris G Foutadakis S Andriopoulou T Spanou VM Droggiti DE Kafousopoulos D Association of Vitamin D with Severity and Outcome of COVID-19: Clinical and Experimental Evidence. J Innate Immun. 2024;16:1–11. 10.1159/000535302 38008066 PMC10764091

[39] Khalil B Sharif-Askari NS Hafezi S Sharif-Askari FS Al Anouti F Hamid Q Vitamin D regulates COVID-19 associated severity by suppressing the NLRP3 inflammasome pathway. PLoS One. 2024;19:e0302818. 10.1371/journal.pone.0302818 38748756 PMC11095707

[40] Mueller AL McNamara MS Sinclair DA Why does COVID-19 disproportionately affect older people? Aging (Albany NY). 2020;12:9959–81. 10.18632/aging.103344 32470948 PMC7288963

[41] Du YN Chen YJ Zhang HY Wang X Zhang ZF Inverse association between systemic immune-inflammation index and bone mineral density in postmenopausal women. Gynecol Endocrinol. 2021;37:650–4. 10.1080/09513590.2021.1885642 33588682

[42] Griffin JS Dent SC Berger SM Pathways linking activity, adiposity, and inflammation to bone mineral density in women and men from NHANES 2007 to 2010. Am J Hum Biol. 2021;33:e23583. 10.1002/ajhb.23583 33645876

[43] Huang Z Xu Z Wan R Hu D Huang Y Associations between blood inflammatory markers and bone mineral density and strength in the femoral neck: findings from the MIDUS II study. Sci Rep. 2023;13:10662. 10.1038/s41598-023-37377-6 37393312 PMC10314938

