# Supplementary material for: Long-term effects of COVID-19 infection on bone mineral density
Source: J Glob Health. 2024 Oct 18;14:05029. doi: 10.7189/jogh.14.05029 (PMC11487469; doi:10.7189/jogh.14.05029)
Supplement: Online Supplementary Document [file jogh-14-05029-s001.pdf]

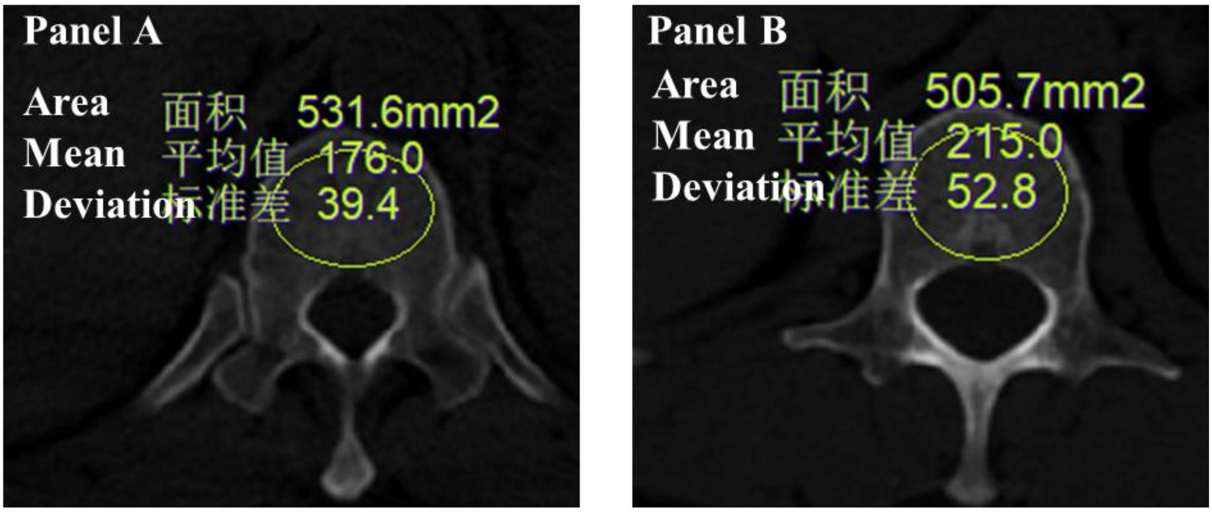

Figure S1. BMD measurement method presentation. BMD was manually measured from the thoracic 12 (**Panel A**) and lumbar 1 (**Panel B**) vertebra using axial chest CT images. The measurement site was placed in the mid-upper part of T12 or L1 (parallel to the pedicle) and between the endplate and the central vascular inlet. The ROI is as large as possible and is located on a homogeneous area that does not include cortical bone. (BMD, bone mineral density; CT, computed tomography, ROI, region of interest)

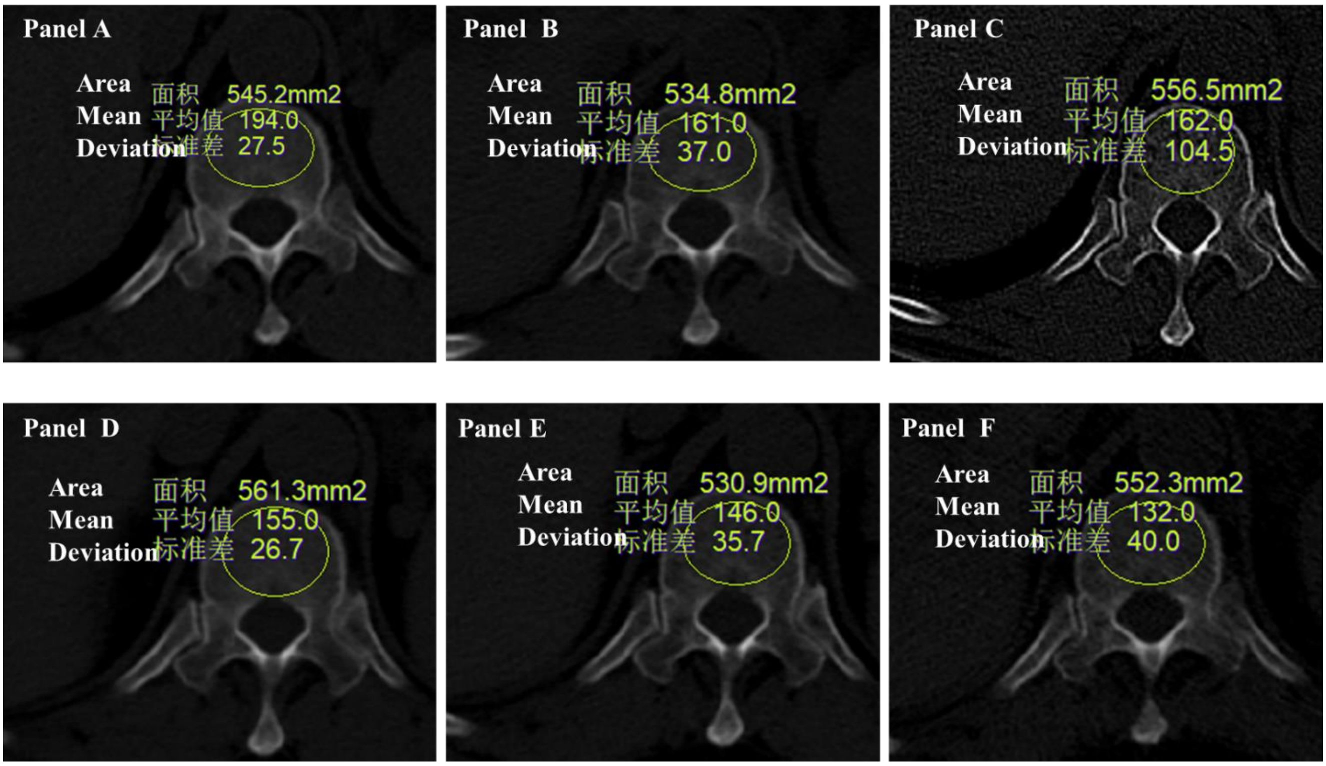

Figure S2. One typical case of early high-rapid decline group. BMD at different time points: day 1(**Panel A**), day 8(**Panel B**), day 32(**Panel C**), day 90(**Panel D**), day 158 (**Panel E**), day 231(**Panel F**). A notable drop of about 60 HU observed by day 231 when compared to the baseline measurement at day 1. (BMD, bone mineral density; HU, Hounsfield units)

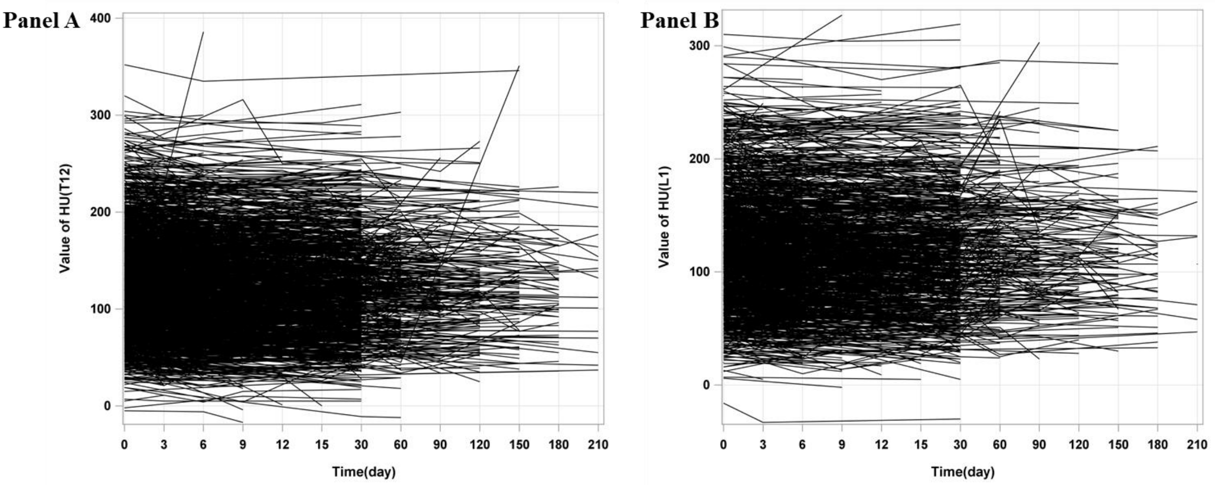

Figure S3. BMD changes were replotted the in the entire population based

on T12 (**Panel A**) and L1 (**Panel B**). (BMD, bone mineral density; L1, lumbar 1; T12, thoracic 12)

Figure S4. BMD changes were replotted in three BMD trajectories based

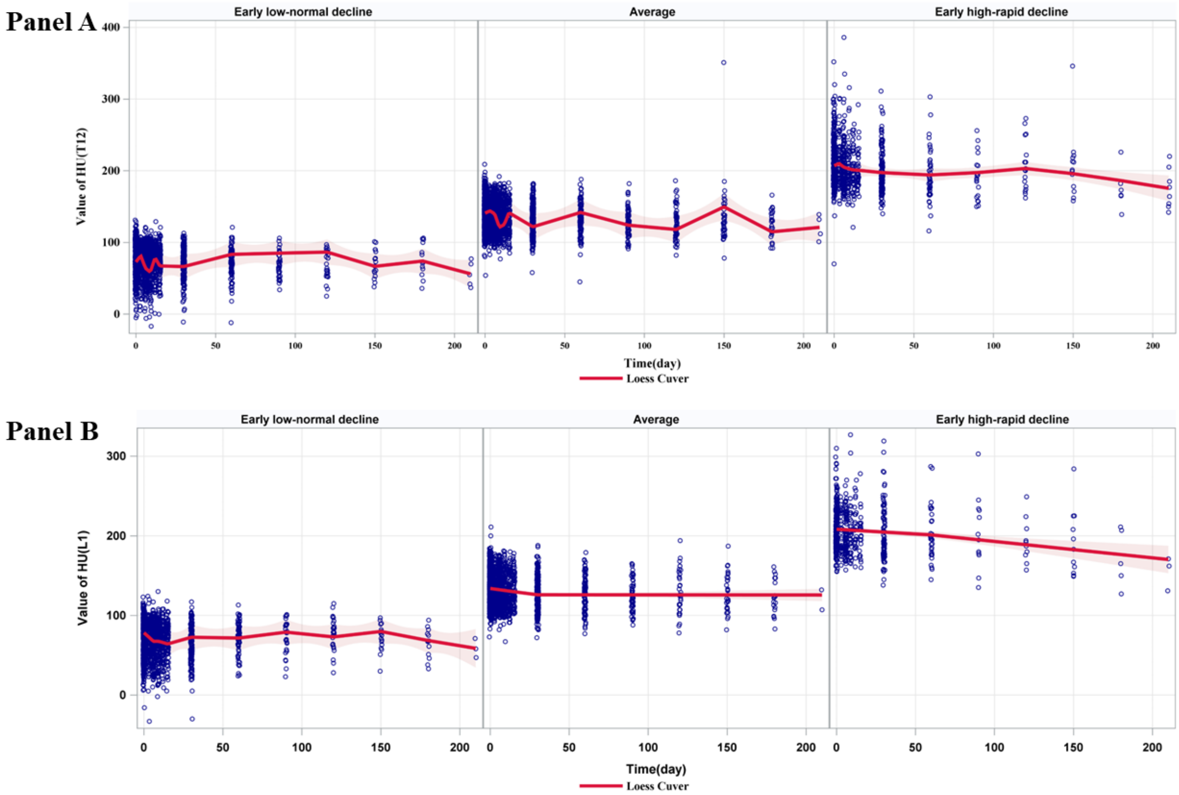

on T12 (**Panel A**) and L1 (**Panel B**). (BMD, bone mineral density; L1, lumbar 1; T12, thoracic 12)

Table S1. Number of patients who underwent CT scans at different time points

	**0d**	**3d**	**6d**	**9d**	**12d**	**15d**	**30d**	**60d**	**90d**	**120d**	**150d**	**180d**	**210d**
**T12**	1767	464	789	448	311	233	591	232	119	95	67	45	17
**L1**	1357	343	584	313	230	181	465	181	94	76	59	37	18

CT, computed tomography; L1, lumbar 1; T12, thoracic 12

Table S2. Risk factors for mortality as identified by multivariate logistic regression (T12)

Risk factor	OR (95%CI)	*p*
**Sex**		
Male	1.00(ref)	
Female	0.82(0.599~1.123)	0.216
**Age (year)**		
≤40	1.00(ref)	
40~60	0.658(0.208~2.088)	0.478
＞60	1.099(0.366~3.299)	0.866
**Comorbidity**		
No	1.00(ref)	
Yes	1.287(0.948-1.748)	0.106
**BMD trajectory pattern(T12)**		
Early low-normal decline	1.00(ref)	
Average	0.805(0.588~1.102)	0.176
Early high-rapid decline	0.584(0.343~0.994)	**0.048**

BMD, bone mineral density; T12, thoracic 12; OR, odds ratio

Table S3. Risk factors for length of hospital stay (＞10 days) as identified by multivariate logistic regression (T12)

Risk factor	OR (95%CI)	*p*
**Sex**		
Male	1.00(ref)	
Female	0.95(0.771~1.171)	0.629
**Age (year)**		
≤40	1.00(ref)	
40~60	1.664(0.837~3.307)	0.147
＞60	1.367(0.693~2.697)	0.367
**Comorbidity**		
No	1.00(ref)	
Yes	3.677(2.940-4.599)	**＜0.001**
**BMD trajectory pattern(T12)**		
Early low-normal decline	1.00(ref)	
Average	0.745(0.597~0.932)	**0.01**
Early high-rapid decline	0.606(0.435~0.844)	**0.003**

BMD, bone mineral density; T12, thoracic 12; OR, odds ratio

Table S4. Risk factors for mortality as identified by multivariate logistic regression (L1)

Risk factor	OR (95%CI)	*p*
**Sex**		
Male	1.00(ref)	
Female	0.826(0.604~1.130)	0.233
**Age (year)**		
≤40	1.00(ref)	
40~60	0.680(0.216~2.145)	0.511
> 60	1.172(0.396~3.464)	0.775
**Comorbidity**		
No	1.00(ref)	
Yes	1.280(0.943-1.738)	0.114
**BMD trajectory pattern(L1)**		
Early low-normal decline	1.00(ref)	
Average	0.801(0.586~1.095)	0.163
Early high-rapid decline	0.589(0.326~1.066)	0.08

BMD, bone mineral density; L1, lumbar 1; OR, odds ratio

Table S5. Risk factors for length of hospital stay (＞10 days) as identified by multivariate logistic regression (L1)

Risk factor	OR (95%CI)	*p*
**Sex**		
Male	1.00(ref)	
Female	0.974(0.791~1.200)	0.805
**Age (year)**		
≤40	1.00(ref)	
40~60	1.615(0.813~3.205)	0.171
> 60	1.383(0.707~2.707)	0.344
**Comorbidity**		
No	1.00(ref)	
Yes	3.662(2.928-4.578)	**＜0.001**
**BMD trajectory pattern(L1)**		
Early low-normal decline	1.00(ref)	
Average	0.924(0.740~1.154)	0.486
Early high-rapid decline	0.602(0.417~0.868)	**0.007**

BMD, bone mineral density; L1, lumbar 1; OR, odds ratio

Table S6. Evaluation indexes of fitting effect of group 2~6 trajectory models (T12)

Model	*Avep%*	Proportions per class%	*BIC*	*△BIC*	E_j
2_11	95.5-93.4	64.1-35.9	-26389	.	0.824
2_22	95.3-93.6	64.2-35.8	-26393	-4	0.824
2_33	95.2-94.0	64.2-35.8	-26387	6	0.827
3_111	94.2-91.9-94.3	36.6-47.1-16.3	-25649	737	0.851
3_222	94.5-91.4-95.1	36.8-46.9-16.3	-25657	-8	0.850
**3_333**	**94.2-91.5-94.9**	**36.4-46.6-16.9**	**-25650**	**7**	**0.850**
3_323	94.3-91.6-94.8	36.6-46.6-16.8	-25648	2	0.850
4_1111	93.1-90.0-93.0-94.2	26.9-41.0-24.9-7.3	-25188	460	0.862
4_2222	93.1-90.2-93.2-94.0	26.9-41.1-24.7-7.3	-25196	-8	0.863
4_3333	93.1-91.3-88.5-94.8	30.9-41.9-18.0-9.3	-25315	-119	0.859
5_11111	91.0-85.9-87.0-92.6-95.2	14.9-28.2-31.3-19.6-6.0	-24957	358	0.839
5_22222	90.5-86.7-87.0-92.3-95.3	15.0-28.5-31.0-19.5-6.0	-24966	-9	0.839
5_33333	90.7-82.3-88.6-93.4-95.4	16.8-21.3-34.1-21.2-6.6	-24896	70	0.842
6_111111	91.1-87.3-88.0-90.7-91.2-94.0	12.3-26.1-31.3-20.0-8.3-2.0	-24792	103	0.856
6_222222	91.8-87.2-85.2-65.5-89.4-95.8	13.0-26.5-30.0-7.6-17.1-5.7	-24758	34	0.825
6_333333	91.8-83.8-67.8-85.6-92.9-96.4	10.9-23.3-13.7-27.7-18.5-5.9	-24737	21	0.812

Avep%, Average posterior probability; BIC, Bayesian information criterion

Table S7. Evaluation indexes of fitting effect of group 2~6 trajectory models (L1)

Model	*Avep%*	Proportions per class%	*BIC*	*△BIC*	E_j
2_11	89.7-92.1	65.0-35.0	-19915	.	0.711
2_22	89.8-92.1	65.0-35.0	-19923	-8	0.711
2_33	89.8-92.1	65.0-35.0	-19925	-2	0.712
3_111	94.5-80.4-95.2	38.7-46.7-14.6	-19290	635	0.749
3_222	94.6-80.4-94.9	38.7-46.7-14.6	-19298	-8	0.749
**3_333**	**94.2-80.5-95.1**	**38.9-46.5-14.6**	**-19300**	**-2**	**0.750**
4_1111	93.3-76.5-91.3-96.9	28.7-39.8-24.3-7.1	-18907	393	0.761
4_2222	93.6-76.4-91.3-96.4	28.9-39.8-24.2-7.1	-18921	-14	0.761
4_3333	94.0-76.4-91.1-96.6	28.6-39.9-24.4-7.2	-18928	-8	0.761
5_11111	92.6-87.8-69.8-92.5-96.3	14.0-29.3-31.2-19.0-6.5	-18654	274	0.751
5_22222	92.4-87.7-69.7-92.4-96.3	14.1-29.5-31.0-18.9-6.5	-18670	-16	0.751
5_33333	91.8-88.2-69.8-92.6-96.5	14.2-29.4-31.1-18.8-6.5	-18679	-9	0.752
6_111111	91.0-88.7-67.9-86.9-90.2-95.1	10.8-25.4-28.8-20.4-9.5-5.2	-18497	182	0.753
6_222222	91.5-88.6-67.8-86.9-91.0-95.3	10.7-25.5-28.7-20.6-9.5-5.1	-18514	-17	0.754
6_333333	90.6-88.8-67.7-86.9-91.2-95.2	10.7-25.5-28.5-20.6-9.6-5.2	-18524	-10	0.754

Avep%, Average posterior probability; BIC, Bayesian information criterion

Table S8. Fitting effect evaluation of each trajectory group in the three-group trajectory model

(T12)

**Trajectory group**	***Avep%***	***OCC***	P_j	π_j
Group 1	94.23	28.5	35.88	36.45
Group 2	91.55	12.4	47.76	46.63
Group 3	94.91	91.5	16.36	16.92

Avep%, Average posterior probability; OCC, Odds of Correct Classification

Table S9. Fitting effect evaluation of each trajectory group in the three-group trajectory model (L1)

**Trajectory group**	***Avep%***	***OCC***	P_j	π_j
Group 1	94.25	25.7	33.33	38.94
Group 2	80.52	4.8	54.61	46.49
Group 3	95.07	113.0	12.05	14.57

Avep%, Average posterior probability; OCC, Odds of Correct Classification
